# Investigating Sensitivity to Auditory Cognition in Listening Effort Assessments: A Simultaneous EEG and Pupillometry Study

**DOI:** 10.1002/brb3.70135

**Published:** 2024-10-31

**Authors:** Samet Kılıç, Eser Sendesen, Filiz Aslan, Nurhan Erbil, Özgür Aydın, Didem Türkyılmaz

**Affiliations:** ^1^ Department of Audiology, Faculty of Health Science Trakya University Edirne Turkey; ^2^ Department of Audiology, Faculty of Health Science Hacettepe University Ankara Turkey; ^3^ Department of Biophysics, Faculty of Medicine Hacettepe University Ankara Turkey; ^4^ Department of Linguistics, Faculty of Languages and History‐Geography Ankara University Ankara Turkey

**Keywords:** auditory attention, auditory cognition, listening effort, pupillometry, working memory

## Abstract

**Background:**

It is still not fully explained what kind of cognitive sources the methods used in the assessment of listening effort are more sensitive to and how these measurement results are related to each other. The aim of the study is to ascertain which neural resources crucial for listening effort are most sensitive to objective measurement methods using differently degraded speech stimuli.

**Methods:**

A total of 49 individuals between the ages of 19 and 34 with normal hearing participated in the study. In the first stage, simultaneous pupillometry, electroencephalogram (EEG), and single‐task paradigm reaction time (RT) measurements were made during the challenging listening and repetition task with noise‐vocoded speech. Two speech reception thresholds (SRT) (50% and 80%) for two vocoding conditions (16 and 6 channels) were collected, resulting in 4 conditions. In the second stage, the Rey Auditory Verbal Learning Test (RAVLT) and the test of attention in listening (TAIL) were applied. Stepwise linear regression analyses were conducted to examine the predictors of listening effort measurements.

**Results:**

A significant difference was found between 6 and 16 channel stimuli in both pupil dilation change and EEG alpha band power change. In the hardest listening condition, whereas RAVLT scores are significant predictors of pupil dilation change, TAIL scores are significant predictors of EEG alpha power. As the stimulus difficulty decreased, the factors that predicted both EEG and pupillometry results decreased. In the single‐task paradigm, a significant regression model could not be obtained at all four difficulty levels.

**Conclusion:**

As a result of the study, it was found that the pupil dilation change was more sensitive to auditory memory skills and the EEG alpha power change was more sensitive to auditory attention skills. To our knowledge, this study is the first to investigate the sensitivity of different listening effort measurement methods to auditory cognitive skills.

## Graphical abstract

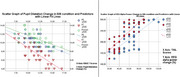

## 1 Introduction

Listening effort, defined as the conscious allocation of mental resources to overcome obstacles to achieving listening goals, is a crucial aspect of auditory processing (Pichora‐Fuller et al. 2016). This definition underscores that effort isn't solely determined by the complexity of the task at hand but also by the active utilization of cognitive abilities to tackle challenges (Winn et al. 2018).

Research indicates that cognitive processing intensifies when individuals process degraded speech, consequently increasing listening effort (Francis and Love 2020; Peelle 2018). Functional magnetic resonance imaging (fMRI) and electroencephalogram (EEG) studies reveal alterations in cognitive processing, reflected in changes in pupil dilation and listener behavior (Gürses et al. 2018; Sendesen et al. 2023).

Although numerous studies explore the role of cognitive resources in understanding acoustically impaired speech, the specific cognitive processes involved remain less understood. Some theories suggest that, in challenging acoustic conditions, listeners may rely on a single cognitive network or employ dissociable processes selectively based on the situation (Rönnberg et al. 2022). Two key components frequently emphasized in this context are working memory and auditory attention (White and Langdon 2021).

Yet, it remains unclear which neural sources are more sensitive to the evaluation methods for measuring listening effort and how these measurements relate to one another. Conducting simultaneous evaluations of listening effort methods is vital to ensuring participants maintain consistent motivation and neural resource levels (Strand et al. 2018). This study aims to ascertain which neural resources crucial for listening effort are most sensitive to objective measurement methods, using differently degraded speech stimuli and accounting for individual cognitive and auditory processing skills.

## 2 Materials and Methods

### 2.1 Participants

A total of 49 individuals with normal hearing participated in the study. Exclusions were made for individuals with vision problems, prior hearing issues, or diagnosed otological, neurological, mental, psychiatric, or other pathologies that could potentially impact the study's tests. Participants were instructed to abstain from medication, coffee, or cigarettes for a minimum of 24 h before their involvement to mitigate potential influences on pupil dilation amplitude.

Pure‐tone hearing thresholds between 125 and 8000 Hz were determined, and individuals with thresholds worse than 15 dB HL at any frequency were not included in the study. During the study, seven individuals were excluded due to artifactual EEG recordings, three individuals were excluded due to excessively noisy pupillometry results, and six individuals did not participate in the second stage. Of the remaining 33 participants aged between 19 and 29, 17 were women. Demographic information for the participants is provided in Table 1.

**TABLE 1:** Demographic information for the participants.

Characteristic	Participants (*n* = 33)
Age (Year) (SD)	23.84 (3,2)
Gender, (%) *n*	
Female	51.5% (17)
Male	48.5% (16)
Education (%) *n*	
Graduate student	60.6% (20)
Graduate	27.3% (9)
Postgraduate	12.1% (4)
Occupation (%) *n*	
Student	75.6% (25)
Research assistant	12.2% (4)
Other	12.2% (4)
Pure tone average (SD)	
Right ear	2.3 (5,6)
Left ear	3.4 (5,9)

Abbreviation: SD, Standard deviation.

### 2.2 Design

Hacettepe University Non‐Interventional Clinical Research Ethics Board gave its approval for this study (GO 20/888). Informed written consent was obtained from all individual participants included in the study. The study was conducted in two distinct sessions. In the initial stage, participants underwent simultaneous pupillometry, EEG recording, and single‐task paradigm reaction time (RT) measurements. These assessments were conducted during a challenging listening and repetition task involving noise‐vocoded speech. In the subsequent stage, administered at the same time of day and under similar fatigue levels as the first stage, participants underwent the Rey Auditory Verbal Learning Test (RAVLT) and the test of attention in listening (TAIL).

The first stage of the study was conducted in an electrically and sound‐isolated laboratory, ensuring a controlled environment with consistent ambient lighting. Conversely, the second‐stage assessments were carried out within a sound booth room.

### 2.3 First Stage

In the first part, EEG and pupillometry recordings were taken simultaneously, and RT measurements were made during the speech stimulus repetition task of four different difficulties presented to the participants. Participants’ head positions were kept constant throughout the test with the support of a chin stabilizer, and eye fixation was achieved by asking them to look at the cursor on the screen in front of them throughout the test. A break was given after the test was completed for each listening condition. Sound stimuli were presented by inserting earphones to prevent the EEG cap electrode from being affected. Participants’ RTs are the time between the end of the sentence presentation and the participant starting to repeat.

### 2.4 Speech Material of the First Stage

Turkish matrix (TM) test sentences were employed for the initial measurements (Zokoll et al. 2015). The order of the words in the TM material is noun + number + adjective + object + verb. Although there are only 50 words in total in TM, it offers the opportunity to evaluate 100,000 different sentences because it allows you to freely combine words among different word groups. The likelihood that the participants will correctly guess the presented sentences is relatively low because they are syntactically appropriate but not semantically appropriate. In this study, 30 lists, each consisting of 20 sentences, were used randomly by the same participant without repetition. At the beginning of the test, the sentences were presented open‐endedly through Sennheiser HDA200 headphones at 0 dB signal‐to‐noise ratio (SNR). Although the noise level used during the test is constant (65 dB SPL), the speech stimulus changes adaptively. As a result of the test, SNRs are obtained from the participants, and they can correctly guess 80% and 50% of the words presented to them.

A total of 60 sentences were selected randomly from our dataset, and their durations were standardized to precisely 3.5 s each. The process of generating noise‐vocoded speech was initiated after synchronization had been established. Custom MATLAB scripts were utilized to process both the sentences and background noise, encompassing TM test noise and multi‐talker babble. The overall frequency range was divided into either 6 or 16 logarithmically spaced channels (Corps and Rabagliati 2020).

Envelope information from each channel was extracted by taking the absolute value from the Hilbert transform. This information was utilized to modulate noise within the same frequency band. The resulting noise bands were then recombined to generate sentences encoded with noise, along with the accompanying background noise. Additionally, the root‐mean‐square values of the sentences and background noise were equalized using MATLAB.

**FIGURE 1:**
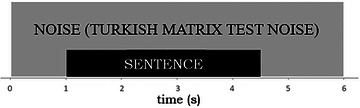
Speech stimulus used in the study.

During the construction of the stimulus, noise was introduced solely 1 s before the commencement of each sentence and persisted for 1.5 s following its conclusion. This allowed for the measurement of alterations in pupil dilation and EEG alpha band activity during instances of strenuous auditory processing. Each stimulus was characterized by a total duration of 6 s, as illustrated in Figure 1, and was employed in the study.

Participants’ individual speech reception thresholds (SRT) were determined using the TM. Two SRTs (50% and 80%) for two vocoding conditions (16 and 6 channels) were collected, resulting in four conditions. These levels were denominated as “8016” for 16‐channel stimuli at an 80% SRT, “806” for 6‐channel stimuli at an 80% SRT, “5016” for 6‐channel stimuli at a 50% SRT, and “506” for 6‐channel stimuli at a 50% SRT. Each participant was exposed to a total of 240 sentences, distributed across these 4 difficulty levels, with an equitable allocation of 60 sentences per level. Following the presentation of each 6‐s stimulus, a 3‐s pause was observed before introducing the subsequent sentence.

Because the signal quality can be decreased without necessarily leading to poor intelligibility, spectral degradation is an ideal aspect of speech signals to investigate with measures of listening effort (Shannon et al. 1995). Pals, Sarampalis, and Başkent (2013) used normal hearing listeners who heard vocoded speech with a variable number of spectral channels to investigate the effect of spectral resolution on listening effort. The vocoded speech was described as “CI simulations” based on the historical performance match between successful CI listeners and normal hearing listeners who were given noise‐vocoded speech (Pals, Sarampalis, and Başkent 2013).

To mitigate potential participant fatigue, a well‐considered 5‐min respite was provided after the conclusion of each difficulty level session. Throughout the speech repetition task, concurrent EEG and pupillometry data were diligently recorded. Furthermore, the time interval between the termination of sentence presentation and the initiation of sentence repetition, constituting the RT for the single‐task paradigm during the test, was measured.

### 2.5 EEG Recording

EEG recording was conducted using a 19‐channel NuAmps II Neuroscan amplifier. Electrodes were positioned on the scalp according to the standard 10–20 configuration, and electrical activity was recorded by placing clip electrodes on both earlobes. Electrode A2, located on the right earlobe, served as the reference electrode during recording, and changes were calculated with respect to this reference.

The impedances of all electrodes were maintained below 5 kΩ during the recording process. Data analysis of the recorded information was performed using EEGlab v14.1.2 within MATLAB 2016a software. EEG data underwent initial filtering within the frequency range of 1–60 Hz, and a notch filter was applied to eliminate 50 Hz artifacts. Ocular artifacts were mitigated using a standard ocular reduction algorithm.

Alpha band amplitude was extracted from EEG data at electrodes P3, P4, and Pz via a bandpass filter set between 8 and 12 Hz. The envelope of each EEG segment obtained was processed using the Hilbert transformation. For each participant, alpha band amplitude was calculated during the resolution period of the presented stimulus (specifically, a 1‐s duration ending 200 ms before the sentence's conclusion). This value was then subtracted from the alpha band amplitude value at the baseline (ranging from 300 to 800 ms after noise onset) and divided by the alpha band amplitude value at the baseline. The resulting value was multiplied by 100 to express the percentage change in alpha band amplitude from baseline to the stimulus resolution period.

### 2.6 Pupillometry

Pupil dilation was measured using the Eyelink 1000+ pupillometry device (SR Research, Ontario, Canada). This system recorded changes in pupil diameter at a sampling rate of 1000 Hz. The pupil diameter waveforms obtained from each trial were processed using R v4.2 software and associated scripts. In order to minimize the potential impact of factors, such as habituation, excitement, or arousal, data analysis excluded the first five stimuli. Pupil diameter values falling below three standard deviations from the mean were identified as eyeblinks and were also excluded. Additionally, eye movements that deviated more than 10° from the fixation target were not included in the analysis. Interpolation was applied from 60 ms before the onset of an eyeblink to 130 ms following the eyeblink event. The average pupil diameter for each participant was assessed during the course of the experiment.

To calculate the maximum pupil size change experienced by participants during the experiment, we used the average pupil diameter during the noise period before the speech stimulus (0–1 s) as the baseline. Subsequently, the average pupil diameter during the stimulus resolution process (2–6 s) was examined to determine each participant's maximum pupil size. This value was calculated by subtracting the average pupil diameter during baseline from the maximum pupil diameter during the resolution process. The resulting value was then divided by the average pupil diameter during the baseline and multiplied by 100 to express the percent change in maximum pupil diameter from the average baseline pupil diameter.

### 2.7 Second Stage

#### 2.7.1 Rey Auditory Verbal Learning Test

RAVLT, originally devised by Rey, serves as a widely recognized tool for evaluating an individual's function of working and long‐term memory, learning, recognition, forgetting, proactive and retroactive interference, temporal order, and motivation (Schmidt 1996). This assessment comprises two lists, A and B, each containing 15 commonplace and concrete words. Participants are tasked with memorizing and subsequently reciting these words. The conventional administration procedure unfolds as follows: List A is verbally presented a total of five times, with immediate recall following each presentation (A_1_ to A_5_). Subsequently, List B, an interference list, is introduced, and participants are required to recall words from this newly presented list (B). Following this interference trial, participants are challenged to recall List A without any preceding presentation (A_6_). Notably, the introduction of List B has a disruptive effect on the subsequent recall of words from List A, a phenomenon known as retroactive interference. For delayed recall, the words in list A are asked to be remembered again after waiting 30 min (A_7_). In this study, the mean of the first five trials (A_mean_), the sixth trial, and the seventh trial of the A list (A_6_ and A_7_) has been used.

### 2.8 Test of Attention in Listening

The TAIL is an auditory attention assessment task that requires participants to judge the similarity or dissimilarity of successively presented tones. These tones display both frequency and spatial location variations (Zhang et al. 2012). Using a button box response mechanism, participants are instructed to indicate whether two tones are “the same” or “different” in terms of either frequency or location.

The tones are presented with an earphone at the participants’ most comfortable loudness level. The frequencies of the tones are arbitrarily chosen between 476 and 2000 Hz, with the spectral gap between any two consecutive tones maintained at a minimum of 2.1 equivalent rectangular bandwidths (Zhang et al. 2012).

TAIL assesses the capacity to selectively focus on a task‐relevant dimension, either frequency or location (in the F condition, frequency was the task‐relevant dimension and location was the distracting dimension, whereas in the L condition, location was the relevant and frequency the irrelevant dimension); RT serves as the primary performance metric in this regard. The test yields two measures per condition (frequency and location) that are computed from the RT data: involuntary orientation (IO) and conflict resolution (CR). IO reflects the RT cost associated with the involuntary shift of attention to the task‐irrelevant dimension, indicating processing efficiency. Conversely, CR measures the RT cost incurred in resolving such conflicts and implies the involvement of executive control mechanisms in the resolution process.

The purpose of this assessment task is to evaluate improvements in auditory sustained attentional control, which refers to the ability to maintain relevant information related to a goal despite the presence of distractions. In this study, F‐IO, F‐CR, L‐IO, and L‐CR scores were used.

### 2.9 Statistical Analyses

In this study, IBM SPSS Statistics 26 software was used for statistical analysis. The mean standard deviation and minimum and maximum values are given as descriptive statistical data. Graphical (histograms, Q–Q plots) and statistical (Shapiro–Wilk Test) methods were applied to evaluate whether the groups had a normal distribution. Whether RT, EEG alpha power, and pupil dilation changes differed according to difficult listening conditions was evaluated with a repeated measures ANOVA test. If there was a significant difference in the ANOVA result, post hoc tests with a Bonferroni‐corrected significance level were used to determine which groups the difference was between. To evaluate the interaction effects of vocoding and SRT on EEG alpha power and pupil dilation, two‐way ANOVA tests were used. The relationship among the TAIL, RAVLT, and listening effort results was examined with Pearson correlation analysis. Stepwise linear regression analyses were conducted to examine the predictors of listening effort measurements, which were RAVLT and TAIL scores that exhibited statistically significant correlations with all listening effort measurements across each challenging listening condition. The margin of error was determined as *p* < 0.05.

## 3 Results

### 3.1 Effect of Different Listening Conditions on Listening Effort

#### 3.1.1 Single‐Task Paradigm

**FIGURE 2:**
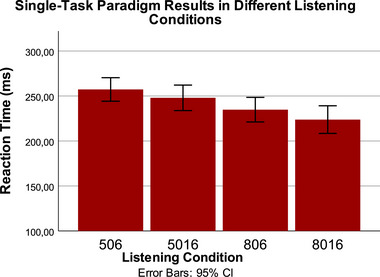
Single‐task paradigm RT results under different difficult listening conditions. Although statistically significant difference was found as a result of the repeated measures of ANOVA test (*p* = 0.03), there wasn't any significant difference found in intra‐group comparisons (*p* > 0.05). RT, reaction time.

Figure 2 shows single‐task paradigm RT results under different difficult listening conditions. The difference between different listening conditions in terms of RT was examined with a repeated measures ANOVA test. A statistically significant difference was found as a result of the ANOVA test (*p* = 0.03), but no significant difference was found in intra‐group comparisons (*p* > 0.05). Although not statistically significant, the largest RT on average across conditions was observed in the 506 condition, which is the most difficult condition.

#### 3.1.2 Pupillometry

**FIGURE 3:**
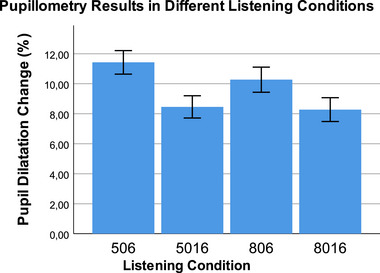
Pupil dilation change results under different difficult listening conditions. The repeated measures of ANOVA result was statistically significant (*p* < 0.001, *F* = 61.48, *η*_p_^2^ = 0.86). When we look at intra‐group comparisons with Bonferroni correction, the highest pupil dilation change was seen in the 506 condition with 6 channels and 50% SRT, and this difference was statistically significant (*p* < 0.001). In intra‐group comparisons, the only match that was not statistically significant was found between the 806 and 8016 conditions with 80% SRT (*p* > 0.05). SRT, speech reception thresholds.

Figure 3 shows pupillometry results under different difficult listening conditions. The difference between different listening conditions in terms of the percentage change in pupil dilation was examined with a repeated measures ANOVA test, and the test result was statistically significant (*p* < 0.001, *F* = 61.48, *η*_p_^2^ = 0.86). When we look at intra‐group comparisons, the highest pupil dilation change was seen in the 506 condition with six channels and 50% SRT, and this difference was statistically significant (*p* < 0.001). In intra‐group comparisons, the only match that was not statistically significant was found between the 806 and 8016 conditions with 80% SRT (*p* > 0.05).

#### 3.1.3 Electroencephalogram

**FIGURE 4:**
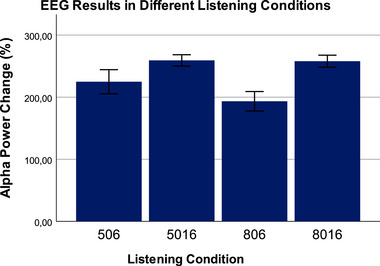
The percentage change in EEG alpha band amplitude results under different difficult listening conditions. The repeated measures of result were found to be statistically significant (*p* < 0.001, *F* = 112.33, *η*_p_^2^ = 0.78). In intra‐group comparisons with Bonferroni correction, the lowest alpha band amplitude change was seen in the 506 condition with 6 channels and 80% SRT, and this difference was found to be statistically significant (*p* < 0.001). In intra‐group comparisons, the only match that was not statistically significant was found between the 5016 and 8016 conditions with 16 channels, 50% SRT, and 80% SRT (*p* > 0.05). EEG, electroencephalogram; SRT, speech reception thresholds.

Figure 4 shows the percentage change in EEG alpha band amplitude results under different difficult listening conditions. The difference between different listening conditions in terms of the percentage change in EEG alpha band amplitude was examined with a repeated measurements ANOVA test. The test result was found to be statistically significant (*p* < 0.001, *F* = 112.33, *η*_p_^2^ = 0.78). Looking at intra‐group comparisons, the lowest alpha band amplitude change was seen in the 506 condition with 6 channels and 80% SRT, and this difference was found to be statistically significant (*p* < 0.001). In intra‐group comparisons, the only match that was not statistically significant was found between the 5016 and 8016 conditions with 16 channels, 50% SRT, and 80% SRT (*p* > 0.05).

### 3.2 SRT and Vocoding

To determine the effect of SRT and vocoding on changes in pupil dilation or alpha power, two‐way ANOVA tests were used. For pupil dilation, there was no significant interaction term (*p* = 0.085). Main effect models indicated significant effects of both SRT (*p* = 0.03) and vocoding (*p* < 0.001). Pupil size was 30% larger in the 6‐channel condition compared to the 16‐channel condition and 10% larger in the 80% SRT condition compared to the 50% SRT condition. For EEG alpha power, there was a significant interaction term (*p* = 0.03). Main effect models indicated significant effects of both SRT (*p* = 0.02) and vocoding (*p* < 0.001). Alpha power change was 20% lower in the 6‐channel condition compared to the 16‐channel condition and 10% lower in the 80% SRT condition compared to the 50% SRT condition.

### 3.3 Relationship Among Auditory Attention, Memory, and Listening Effort Measurements

The relationship among single‐task paradigm RTs, pupil dilation percentage change, and EEG alpha amplitude change percentage values obtained in listening conditions at four different difficulty levels and TAIL and RAVLT results were examined with Pearson correlation analyses. All correlation analysis results are shown in Table 2.

**TABLE 2:** Pearson correlation analyses between listening effort measures, test of attention in listening (TAIL) and Rey Auditory Verbal Learning Test (RAVLT) scores.

	p506		a506		r506	
	*r*	*p*	*r*	*p*	*r*	*p*
**A_mean_**	−0.77***	< 0.001	−0.61***	< 0.001	−0.41*	0.02
**A_6_**	−0.75***	< 0.001	−0.64***	< 0.001	−0.34*	0.04
**A_7_**	−0.67***	< 0.001	−0.58**	0.002	−0.21	> 0.05
**F‐IO**	0.58**	0.004	0.81***	< 0.001	0.31*	0.04
**F‐CR**	0.54**	0.004	0.56**	0.003	0.33	> 0.05
**L‐IO**	0.61**	0.002	0.75***	< 0.001	0.41	> 0.05
**L‐CR**	0.47*	0.01	0.6**	0.002	0.33	> 0.05

	**p5016**		**a5016**		**r5016**	
	***r***	***p***	***r***	***p***	***r***	***p***
**A_mean_**	−0.69***	< 0.001	−0.54**	0.002	−0.34*	0.04
**A_6_**	−0.67***	< 0.001	−0.67***	< 0.001	−0.4*	0.02
**A_7_**	−0.66***	< 0.001	−0.56**	0.002	−0.31	> 0.05
**F‐IO**	0.61***	< 0.001	0.78***	< 0.001	0.36*	0.04
**F‐CR**	0.55**	0.003	0.47**	0.01	0.23	> 0.05
**L‐IO**	0.57**	0.007	0.74***	< 0.001	0.35*	0.04
**L‐CR**	0.61**	0.002	0.56**	0.04	0.37*	0.03

	**p806**		**a806**		**r806**	
	***r***	***p***	***r***	***p***	***r***	***p***
**A_mean_**	−0.72***	< 0.001	−0.6***	< 0.001	−0.32	> 0.05
**A_6_**	−0.69***	< 0.001	−0.62***	< 0.001	−0.35*	0.04
**A_7_**	−0.71***	< 0.001	−0.61***	< 0.001	−0.35*	0.04
**F‐IO**	0.65***	< 0.001	0.77***	< 0.001	0.41*	0.02
**F‐CR**	0.58**	0.003	0.55***	< 0.001	0.45*	0.01
**L‐IO**	0.63***	< 0.001	0.69***	< 0.001	0.43*	0.01
**L‐CR**	0.56**	0.008	0.55***	< 0.001	0.31*	0.04

	**p8016**		**a8016**		**r8016**	
	***r***	***p***	***r***	***p***	***r***	***p***
**A_mean_**	−0.61***	< 0.001	−0.56**	0.001	−0.33	> 0.5
**A_6_**	−0.62***	< 0.001	−0.54**	0.001	−0.27	> 0.5
**A_7_**	−0.58**	0.001	−0.54**	0.002	−0.22	> 0.5
**F‐IO**	0.6***	< 0.001	0.58**	0.001	0.32	> 0.5
**F‐CR**	0.56**	0.003	0.5**	0.003	0.33	> 0.5
**L‐IO**	0.63***	< 0.001	0.61***	< 0.001	0.24	> 0.5
**L‐CR**	0.46*	0.02	0.47*	0.03	0.29	> 0.5

*Note: r* values of the correlations are listed. p: pupil dilation change, a: EEG alpha power change, r: single‐task paradigm response time, “8016” for 16‐channel stimuli at an 80% speech reception threshold (SRT), “806” for 6‐channel stimuli at an 80% SRT, “5016” for 6‐channel stimuli at a 50% SRT, and “506” for 6‐channel stimuli at a 50% SRT, A_mean_: mean of the first 5 trials of Rey Auditory Verbal Learning Test (RAVLT), A_6_: 6th trial of the RAVLT, A_7_: 7th trial of AVLT, F‐IO: frequency condition—involuntary orienting of test of attention in listening (TAIL), F‐CR: frequency condition—conflict resolution of TAIL, L‐IO: location condition—involuntary orienting of TAIL, L‐CR: location condition—conflict resolution of TAIL.

* *p* < 0.05.

** *p* < 0.01.

*** *p* < 0.001.

For the cognitive data that had a significant relationship and met other multiple linear regression analysis conditions, a multiple stepwise linear regression analysis was utilized to see how well it could predict the results of the listening effort assessments. The regression analysis results are shown in Table 3. In the single‐task paradigm, a significant regression model could not be obtained at all four difficulty levels.

**TABLE 3:** Stepwise linear regression analyses for predicting factors of listening effort measurements.

		β	SE	Standardized β	*t*	*p* value	95% Confidence interval		
							Lower bound	Upper bound	
**p506**	Constant	22.998	1.755		13.103	< 0.001***	19.408		26.588
	**A_mean_**	−0.523	0.124	−0.573	−4.237	< 0.001***	−0.776		−0.271
	**A_6_**	−0.401	0.179	−0.275	−2.241	0.002**	−0.766		−0.035
	**A_7_**	−0.327	0.185	−0.225	−1.924	0.033*	−0.706		−0.051
**a506**	Constant	273.763	37.167		7.366	< 0.000***	197.503		350.022
	**F‐IO**	0.165	0.189	0.569	5.285	< 0.000***	0.382		1.339
	**L‐IO**	0.206	0.101	0.316	3.228	0.003**	0.306		0.913
**p5016**	Constant	16.023	2.681		5.976	< 0.001***	10.547		21.499
	**A_6_**	−0.616	0.114	−0.698	−5.425	< 0.000***	−1.009		−0.0265
	**A_mean_**	−0.464	0.167	−0.336	−2.225	0.001**	−0.808		−0.031
**a5016**	Constant	293.344	23.648		12.404	< 0.001***	244.822		341.867
	**F‐IO**	0.640	0.165	0.539	4.115	< 0.001***	0.434		0.923
	**L‐IO**	0.542	0.232	0.466	3.588	< 0.001**	0.194		0.764
	**A_mean_**	−7.147	1.737	−0.452	−3.115	< 0.001***	−10.71		−3.584
**p806**	Constant	18.853	1.409		13.377	< 0.001***	15.975		21.731
	**A_6_**	−0.868	0.163	−0.796	−7.614	< 0.001***	−1.101		−0.635
	**A_7_**	−0.574	0.189	−0.417	−3.185	< 0.001***	−0.761		−0.377
**a806**	Constant	206.107	46.189		4.462	< 0.001***	111.777		300.437
	**F‐IO**	1.291	0.233	0.636	5.551	< 0.001***	0.116		0.935
	**A_mean_**	−9.255	3.353	−0.316	−2.76	0.01*	−16.103		−2.407
**p8016**	Constant	14.978	0.71		21.098	< 0.001***	13.53		16.426
	**A_7_**	−0.9	0.092	−0.87	−9.824	< 0.001***	−1.086		−0.713
**a8016**	Constant	395.31	13.561		29.15	< 0.001***	367.615		423.006
	**A_7_**	−7.52	1.097	−0.596	−6.854	< 0.001***	−9.761		−5.279
	**A_6_**	−8.031	1.568	−0.445	−5.121	< 0.001***	−11.234		−4.828

*Note*: Unstandardized coefficients *β* values are listed. p: pupil dilation change, a: EEG alpha power change, “8016” for 16‐channel stimuli at an 80% speech reception threshold (SRT), “806” for 6‐channel stimuli at an“ 80% SRT, ”“5016” for 6‐channel stimuli at a 50% SRT, and “506” for 6‐channel stimuli at a 50% SRT; A_mean_: mean of the first 5 trials of Rey Auditory Verbal Learning Test (RAVLT), A_6_: 6th trial of the RAVLT, A_7_: 7th trial of AVLT, F‐IO: frequency condition—involuntary orienting of Test of Attention in Listening (TAIL), L‐IO: location condition—involuntary orienting of TAIL.

* *p* < 0.05.

** *p* < 0.01.

*** *p* < 0.001.

**FIGURE 5:**
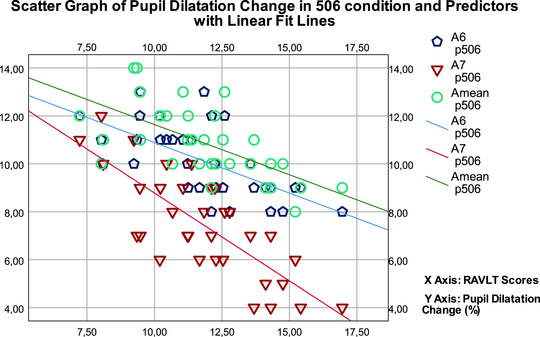
Scatter graph of pupil dilation change in 506 condition and its predictors in the stepwise linear regression model.

**FIGURE 6:**
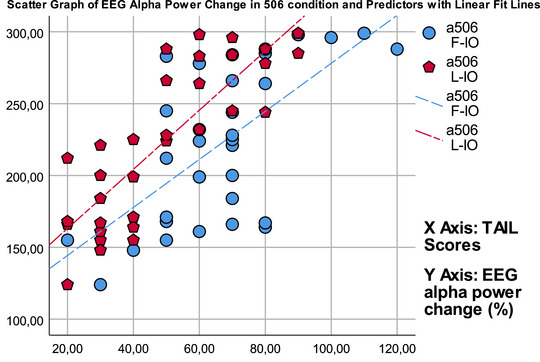
Scatter graph of EEG alpha power change in 506 condition and its predictors in the stepwise linear regression model. EEG, electroencephalogram.

Considering the 506 condition, it was determined that A_mean_, A_6_, and A_7_ were significant predictors in the model created for pupil dilation change. The strongest predictor was found to be A_mean_ (*F* (3,29) = 24.584, *p* < 0.001, with an adjusted *R* ^2^ of 0.718). Figure 5 illustrates a scatter graph of pupil dilation change and its predictors in the stepwise linear regression model. When the EEG alpha band power change was examined, it was determined that F‐IO and L‐IO were significant predictors, and the strongest predictor was F‐IO (*F* (2,30) = 43.899, *p* < 0.001, with an adjusted *R* ^2^ of 0.87). Figure 6 illustrates a scatter graph of EEG alpha power change and its predictors in the stepwise linear regression model.

Considering the 5016 condition, it was determined that A_6_ and A_mean_ were significant predictors in the model created for pupil dilation change. The strongest predictor was found to be A_6_ (*F* (2,30) = 17.782, *p* < 0.001, with an adjusted *R* ^2^ of 0.469). When the EEG alpha band power change was examined, it was determined that F‐IO, L‐IO, and A_mean_ were significant predictors, and the strongest predictor was F‐IO (*F* (3,29) = 21.447, *p* < 0.001, with an adjusted *R* ^2^ of 0.762).

Considering the 806 condition, it was determined that A_6_ and A_7_ were significant predictors in the model created for pupil dilation change. The strongest predictor was found to be A_6_ (*F* (2,30) = 49.023, *p* < 0.001, with an adjusted *R* ^2^ of 0.75). When the EEG alpha band power change was examined, it was determined that F‐IO and A_mean_ were significant predictors, and the strongest predictor was F‐IO (*F* (2,30) = 33.289, *p* < 0.001, with an adjusted *R* ^2^ of 0.669).

Considering the 8016 condition, it was determined that A_7_ was the only significant predictor in the model created for pupil dilation change. (*F* (1,31) = 96.518, *p* < 0.001, with an adjusted *R* ^2^ of 0.749). When the EEG alpha band power change was examined, it was determined that A_6_ and A_7_ were significant predictors, and the strongest predictor was A_7_ (*F* (2,30) = 77.536, *p* < 0.001, with an adjusted *R* ^2^ of 0.827).

## 4 Discussion

In this study, it was examined which auditory cognitive skills crucial for listening effort are most sensitive to objective measurement methods, using differently degraded speech stimuli (noise‐vocoded speech) and accounting for individual auditory attention and memory skills. As a result of the study, it was found that the pupil dilation change was more sensitive to auditory attention skills and the EEG alpha power change was more sensitive to auditory memory skills. To our knowledge, this study is the first to investigate the sensitivity of different listening effort measurement methods to cognitive skills.

In their 2017 study, Miles et al. used noise‐vocoded speech as the speech stimulus, just like in this study, in 4 different difficulties with 6 to 16 channels and 50% to 80% SRT (Miles et al. 2017). A significant difference was found between 6 and 16 channel stimuli in both pupil dilation change and EEG alpha band power change. It has been determined that the effort increases as the number of channels decreases. It was determined that these results were compatible with the findings obtained in this study.

In this study, single‐task paradigm RT results, which is one of the behavioral measurement methods for evaluating listening effort, were obtained. No significant difference was found among RTs at different difficulty levels. When listening effort studies conducted with the single‐task paradigm were examined in the literature, it was reported that noise was more effective in the single‐task paradigm and that SNR affected the task more than effort (Gagne, Besser, and Lemke 2017; Giuliani, Brown, and Wu 2021; Pals et al. 2015). In this study, although the noise intensity remained constant, speech stimuli with two different SRTs of 50% and 80% were used, but unlike previous studies, no significant difference was found in terms of RTs between SRTs in this study. It is thought that the reason for this incompatibility may be the use of individual SRTs instead of standard SNRs.

This study focused on assessing auditory attention and memory as cognitive abilities. The reason for this is that two of the more compelling suggestions in the literature are that acoustically degraded speech requires listeners to rely to a greater extent on verbal working memory and auditory attention (Peelle 2018; Rönnberg et al. 2014; Wingfield, Amichetti, and Lash 2015). Moreover, Francis and Love (2020) indicates that the characterization of listening effort has traditionally focused on the allocation of limited cognitive resources, such as selective attention and working memory (Francis and Love 2020).

TAIL provides information about IO and CR, two outcomes of auditory attention. As a result of the multiple regression analyses performed in this study, it was found that IO was a significant predictor in both frequency and location conditions in the 506 and 806 conditions, and the frequency condition was a significant predictor in the 8016 condition. Although CR had a significant correlation with listening effort measurements, it was not found to be a significant predictor in the regression models, according to Attention Network Review (Petersen and Posner 2012). The fact that parietal electrodes were used to determine EEG alpha power in this study may explain why IO is a significant predictor. Further studies need to be conducted on this subject. According to the conflict‐monitoring view, conflicts in information processing or decision‐making activate the anterior cingulate cortex, which then recruits lateral frontal areas to resolve conflicts (Schmidt 2019). The TAIL test uses pure tones as stimuli. It may be recommended to conduct further studies by determining an attention outcome, including verbal working memory, using auditory attention tests that use speech stimuli as stimuli.

RAVLT is a test battery frequently used for a comprehensive evaluation of memory. Memory is one of the important neural resources in the formation of listening effort. Amichetti et al. (2013) and Picou, Ricketts, and Hornsby (2013) found that participants with low verbal working memory scores made more effort while listening. In this study, a negative relationship was observed between all RAVLT scores and listening effort. In the multiple regression analysis, the change in pupil dilation showed a significant relationship with the A_mean_, A_6_, and A_7_. A_mean_ score is a free recall score related to short‐term memory. A_6_ score is a recall score after the presentation of the distractor list. A_6_ is related to verbal working memory. A_7_ is a delayed recall related to long‐term memory (De Wit et al. 2022). Miller, Gross, and Unsworth (2019), in their study with 138 young adults, recorded pupillometry during long‐term memory and working memory tests. Participants with higher working memory capacity were more successful in the long‐term recall test, and the change in pupil dilation increased less than those with lower working memory capacity. These results are compatible with the results of our study. All of the RAVLT results were significant in estimating pupil dilation change in the listening condition that was the hardest, 506, but only A_7_ was significant in the condition that was the easiest, 8016. These results are consistent with previous studies showing the effect of verbal working memory on listening effort. Multiple regression analyses showed that TAIL scores were more significant predictors than RAVLT in predicting EEG alpha power. However, A_mean_ and A_7_ were included as significant factors in the regression models in other conditions except the 506 condition. Berger et al. (2014) found a significant relationship between long‐term memory and alpha power in their study and showed that semantic long‐term memory is related to alpha power (Berger et al. 2014). These results are compatible with the results of our study.

In a study conducted by McMahon et al. (2016), it was demonstrated that there exists a correlation between the modulation of EEG alpha level and pupil dilation in the context of 16‐channel vocoded speech. However, when examining the conditions of more challenging six‐channel vocoded speech, the relationship between these two variables became considerably less discernible. In a subsequent study, Miles et al. (2017) conducted research with the objective of examining the impact of intelligibility. Their findings revealed that, in contrast to the outcomes obtained from EEG measurements, pupil dilation exhibited a correlation with scores of intelligibility. The same type of speech stimulus was used in this study. When the difficulty level of the speech stimulus increased, memory became more effective in predicting pupil dilation results, whereas the IO of attention was more effective in predicting EEG alpha power. As the stimulus difficulty decreased, this separation decreased, and the factors that predicted both EEG and pupillometry results decreased. These results can be explained by the decrease in the need for neural resources as listening effort decreases (Kiliç, Yiğit, and Türkyilmaz 2022).

### 4.1 Limitations and Future Directions

It is important to recognize a number of limitations that may restrict how broadly we can apply our findings, even if this study offers insightful information about the relationship between cognitive resources and listening effort during demanding auditory tasks.

The very uniform demographics of the subjects, all of whom had normal hearing, pose a constraint for this study. Future studies ought to focus on including a more varied sample, one that includes people with different hearing impairments, ages, and cultural backgrounds. This variety would enable a deeper comprehension of how listening effort and cognitive resources interact in various settings.

The noise‐vocoded speech stimuli employed in our investigation had controlled levels of difficulty. This offered a methodical technique, but it might not have adequately captured the complexity of actual listening situations. Future research should think about including a larger variety of inputs, such as ambient noise and natural speech, to better mimic the intricacies of everyday communication.

In this study, only the alpha‐band power change of the EEG was analyzed. In future studies, researchers can investigate the relationship between pupillometry results and changes in the power of other bands, like beta and theta, which are thought to be related to listening effort.

Another limitation of this study includes analyzing listening effort with EEG alpha power change using the parietal electrodes. The majority of the aforementioned studies described the enhanced alpha activity in this region; it may be assessed using additional electrodes in adjacent regions. Future studies may wish to consider employing a comprehensive analysis of the entire head when extracting the alteration in alpha power during the difficult task of listening.

Our single‐task paradigm results show that RT may not be a sensitive indication of listening effort. A future study could investigate other behavioral measures or task designs for a more precise evaluation of listening effort, including incorporating real‐time feedback to monitor cognitive load.

Memory and focus were evaluated largely using the RAVLT and the TAIL. Increasing the variety of cognitive measures, such as tests of working memory and executive functioning, can provide a more complete picture of the cognitive resources required for listening.

Our research revealed that pupillometry was more responsive to auditory attention skills, but EEG alpha band power fluctuations were linked to auditory memory skills. Future research could investigate the precise neurological mechanisms underlying these discoveries in order to improve clinical and therapeutic tactics.

## 5 Conclusion

This study aimed to investigate the intricate relationship between listening effort and cognitive resources in the context of processing acoustically distorted speech stimuli. The objective of this study was to elucidate the cognitive mechanisms implicated in the distribution of cognitive resources during challenging auditory tasks, specifically in the context of noise‐vocoded speech.

The results of our study underscore the susceptibility of various neural assets to objective measurement methodologies. The utilization of pupillometry has been identified as a valuable measure for assessing auditory memory abilities, whereas alterations in EEG alpha band power have demonstrated a strong association with auditory attention capabilities. The implications of these findings have practical significance in the assessment of listening effort. They indicate that a comprehensive understanding of the cognitive processes underlying listening effort can be achieved by employing a multi‐method approach that integrates pupillometry and EEG measurements.

As the level of stimulus difficulty escalated, there was an observed increase in the predictive power of memory on pupil dilation outcomes. Conversely, attentional IO emerged as a more influential predictor of alterations in EEG alpha power. This observation illustrates the flexible nature of cognitive resource allocation, which adjusts in response to the requirements of the listening task.

Furthermore, a noteworthy association was observed between auditory attention and memory abilities, as well as listening effort. Individuals who achieved higher scores on cognitive tests such as the RAVLT and the TAIL demonstrated more effective allocation of cognitive resources when faced with difficult listening conditions. This underscores the importance of both short‐term and long‐term memory, as well as attentional control, in shaping auditory perception.

This research contributes to our comprehension of listening effort and its cognitive foundations. The findings provide a foundation for future research that can inform clinical practice, technology development, and strategies to improve communication in challenging listening situations, despite their limitations.

## Author Contributions

**Samet Kılıç**: conceptualization, investigation, writing–original draft, methodology, writing–review and editing, software, data curation, formal analysis, validation, resources. **Eser Sendesen**: methodology, formal analysis, investigation, validation. **Filiz Aslan**: methodology, data curation, validation. **Nurhan Erbil**: conceptualization, software, methodology, data curation, validation, supervision, visualization, resources, formal analysis. **Özgür Aydın**: methodology, software, validation, investigation, formal analysis, visualization, supervision. **Didem Türkyılmaz**: methodology, conceptualization, validation, supervision, project administration, resources, funding acquisition.

## Ethics Statement

All procedures performed in studies involving human participants were in accordance with the ethical standards of the institutional and national research committee and with the 1964 Helsinki Declaration and its later amendments or comparable ethical standards.

## Conflicts of Interest

The authors declare no conflicts of interest.

### Peer Review

The peer review history for this article is available at https://publons.com/publon/10.1002/brb3.70135.

## Data Availability

The datasets generated during and/or analyzed during the current study are available from the corresponding author on reasonable request.
